# Modulatory Role of *Moringa Oleifera*-Loaded Silver Nanoparticles on UCP1 and PPARGC1A Genes Expression in an Obesity Rat Model

**DOI:** 10.1007/s12010-025-05511-x

**Published:** 2026-01-23

**Authors:** Omnia Aly, Ammar S. Al Khafaji, Amal F. Gharib, Hend M. Ahmed, Samar Helmy, Nabil A. Shoman, Mohamed M. Hafez

**Affiliations:** 1 Department of Medical Biochemistry, National Research Centre, Cairo, Egypt; 2 College of Pharmacy, Al Zahrawi University, Karbala, Iraq; 3 Department of Clinical Laboratory Sciences, College of Applied Medical Sciences, Taif University, Taif, Saudi Arabia; 4 Department of Biochemistry, Faculty of Pharmacy, Ahram Canadian University (ACU), 6th October, Giza Egypt; 5 Department of Pharmaceutics and Pharmaceutical Technology, Faculty of Pharmacy, Ahram Canadian University, 6th October, Giza Egypt

**Keywords:** Obesity, *Moringa oleifera*, Silver nanoparticles, UCP1, PPARGC1A

## Abstract

Obesity remains a global health crisis, with limited therapeutic options for addressing metabolic dysfunction and inflammation. This study evaluates the therapeutic potential of *Moringa Oleifera*-loaded silver nanoparticles (MO-AgNPs) in modulating obesity-related biochemical and genetic markers. To optimize the synthesis of MO-AgNPs, Response Surface Methodology (RSM) using Design Expert software was employed to identify the optimal conditions for minimizing particle size, polydispersity index, and maximizing zeta potential. Male albino rats with high-carbohydrate, high-fat diet (HCHFD)-induced obesity were treated with AgNPs, *Moringa Oleifera* extract, or MO-AgNPs. Key assessments included biochemical markers, lipid profiles, adipokines, and the expression of thermogenic and metabolic genes (UCP1, PPARGC1A), along with histopathological evaluation of liver and pancreatic tissues. Transmission electron microscopy revealed spherical particles with an average size of approximately 40 nm, consistent with dynamic light scattering data. Treatment with MO-AgNPs significantly enhanced insulin sensitivity, improved lipid profiles, and regulated adipokine levels, while upregulating UCP1 and PPARGC1A expression more effectively than other treatments. Histological analysis demonstrated substantial restoration of liver and pancreatic tissue architecture, highlighting the protective effects of MO-AgNPs. Overall, MO-AgNPs represent an innovative therapeutic strategy, combining the bioactive properties of *Moringa Oleifera* with the enhanced functionality of silver nanoparticles, showing strong potential for addressing obesity and its associated metabolic complications.

**Graphical Abstract:**

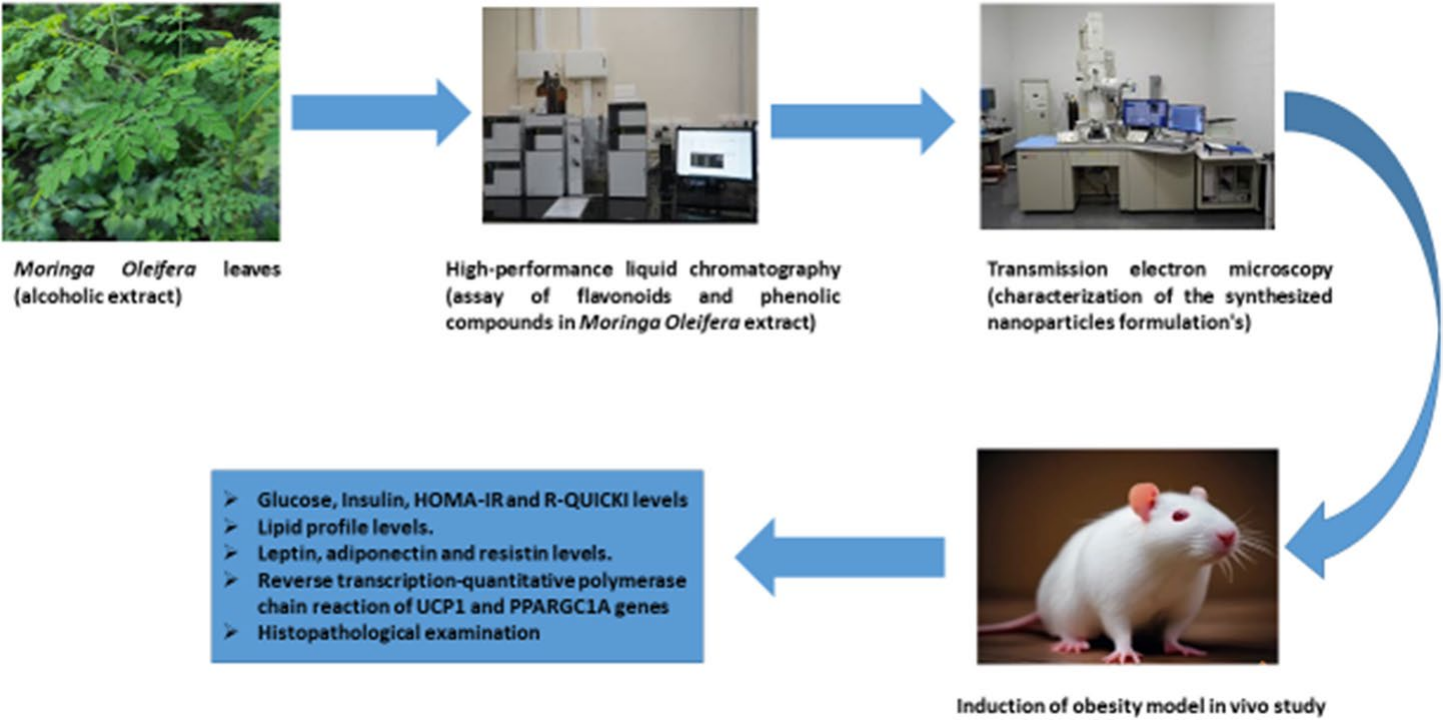

**Supplementary Information:**

The online version contains supplementary material available at 10.1007/s12010-025-05511-x.

## Introduction

Globally, obesity is a serious and immediate threat to public health since it contributes significantly to the burden of death and disability. Obesity is not merely a condition of being overweight; it is a systemic inflammatory disease linked to various metabolic disorders, including chronic kidney disease, diabetes, cancer, insulin resistance, and cardiovascular problems. Adipose tissue (AT), which is made up of adipocytes and stromal vascular cells, plays an important role in the development of metabolic disorders and obesity. The onset of obesity and related diseases is mostly attributed to sedentary lifestyles and insufficient physical activity. Adipose tissue releases a variety of adipokines that significantly impact glucose and lipid metabolism, and these are implicated in the pathophysiology of several diseases [1].

One of the most significant factors in the pathophysiology of obesity is the production of cytokines by adipose tissue, or adipokines. Key adipokines include resistin, adiponectin, and leptin [2]. Leptin, a classic adipokine, attaches to its receptor in the brain to enhance energy expenditure and suppress appetite [3]. Adiponectin plays a significant role in enhancing insulin sensitivity by inducing the phosphorylation of 5′ AMP-activated protein kinase, which in turn promotes glucose absorption into cells by accelerating the translocation of glucose transporter type 4. Insulin resistance and metabolic abnormalities associated with obesity are thought to be influenced by resistin. Understanding the production and regulation of these adipokines is essential for effective risk assessment of obesity-related conditions, including metabolic syndrome, type II diabetes, atherosclerosis, heart disease, and cancer [4].

Adipose tissue also secretes another hormone called resistin, an adipokine that promotes inflammation and is known to be antagonistic to insulin. Although resistin is expressed in various tissues such as preadipocytes, endothelial cells, and vascular smooth muscle cells, its highest expression is found in macrophages [5].

Unlike white adipose tissue (WAT), brown adipose tissue (BAT) is primarily responsible for adaptive thermogenesis. Brown adipocytes’ ability to undergo thermogenesis is largely dependent on their high mitochondrial content and uncoupling protein 1 (UCP1) expression. When active, UCP1 facilitates the release of chemical energy by uncoupling the oxidation of mitochondrial substrates from ATP synthesis, generating heat [6].

Emerging evidence highlights the role of peroxisome proliferator-activated receptor gamma coactivator 1 alpha (PPARGC1A) in lipid metabolism and glucose homeostasis. PPARGC1A is crucial for lipid metabolism, functioning either directly or indirectly. It activates the farnesoid X receptor (FXR), which lowers liver triglyceride levels. Additionally, it regulates the activity of the lipid metabolism-related human hepatic lipase gene (LIPC). PPARGC1A’s expression in the liver and its nuclear receptor, hepatocyte nuclear factor 4α (HNF4α), elevates several apolipoproteins involved in the metabolism of triglycerides and very low-density lipoproteins (VLDLs). In skeletal muscle and pancreatic beta cells, PPARGC1A is crucial for glucose transport. It also co-activates the cholesterol 7-α-hydroxylase (CYP7A1) gene, essential for cholesterol metabolism [7].

Adipose, liver, and muscle metabolically active tissues that are essential to the pathophysiology of obesity and provide direct information on metabolic regulation. However, because peripheral blood is accessible, minimally invasive, and appropriate for large-scale or longitudinal studies, we chose it as the tissue source for gene expression profiling. Systemic inflammatory and immunological responses, which are increasingly identified as major causes of obesity and associated comorbidities, are also reflected in blood [8].

Despite available therapies, most pharmacological approaches fail to address the root causes of obesity, often resulting in limited efficacy and adverse effects. This limitation has led to growing interest in plant-based treatments, which are often more affordable, safer, and possess bioactive properties that can positively influence molecular pathways, and the expression of genes and proteins involved in obesity. Antioxidants like polyphenols have been used as preventive agents to manage the comorbidities of obesity, combating the oxidative stress commonly associated with it. For this reason, polyphenols could be utilized to develop safe and effective anti-obesity medications [9].

Recent scientific advancements have also highlighted the therapeutic potential of plant-based products due to their significant pharmacological efficacy, minimal toxicity, and economic feasibility in comparison to synthetic treatments [10, 11]. Among these, *Moringa oleifera*, known for its vast medicinal potential, has become a focal point due to its nutritional value, use in livestock feed, and therapeutic properties. Often referred to as the “miracle tree,” Moringa boasts a variety of therapeutic qualities attributed to its roots, leaves, and seeds. Its pharmacological properties include anti-inflammatory, anti-cancer, antioxidant, neuroprotective, liver disease prevention, hypoglycemic, hypocholesterolemic, and blood lipid-lowering effects. *Moringa oleifera’s* high concentration of phytochemicals, including flavonoids, glucosinolates, isothiocyanates, and phenolic acids, directly contributes to its beneficial properties [12]. Notably, several individual phytochemicals such as curcumin [13], resveratrol [14], and green tea catechins [15] have demonstrated anti-obesity potential. However, *Moringa oleifera* provides a distinct advantage through its exceptionally diverse and synergistic phytochemical profile, particularly flavonoids (e.g., rutin, quercetin), phenolic acids (e.g., chlorogenic acid), and glucosinolates, which collectively exert antioxidant, anti-inflammatory, lipid-lowering, and antihyperglycemic effects. This multi-targeted mechanism may offer superior therapeutic efficacy compared with single-phytochemical approaches [16, 17].

Despite the promising potential of plant-based therapies, their effectiveness is often limited by poor bioavailability and inconsistent therapeutic outcomes. Nanotechnology provides a powerful strategy to address these challenges by enhancing the stability, delivery, and bioactivity of natural compounds. Beyond improving pharmacokinetics, nanocarriers can synergize with phytochemicals by acting not only as delivery vehicles but also as active modulators, thereby amplifying their therapeutic efficacy [18]. Among these, silver nanoparticles (AgNPs) have gained recognition for their ability to modulate gene expression, reduce oxidative stress, and improve insulin sensitivity. Importantly, AgNPs themselves exert biological effects relevant to obesity, including enhancement of insulin sensitivity through GLUT4 translocation [19], reduction of oxidative stress, and suppression of pro-inflammatory cytokines [20], thereby contributing to improved metabolic regulation. However, their potential toxicity remains an important consideration. Chemically synthesized AgNPs often possess reactive metallic surfaces that can induce oxidative stress, DNA damage, and protein dysfunction [21]. In contrast, green-synthesized AgNPs prepared using plant extracts represent a safer alternative. The phytochemicals present in the extracts act as reducing and capping agents, forming a biocompatible shell around the nanoparticle core. This biogenic coating mitigates oxidative stress and inflammatory responses, thereby significantly lowering cytotoxicity while retaining strong antibacterial and therapeutic efficacy [22]. Furthermore, green synthesis offers additional advantages, including reduced use of hazardous solvents, minimized environmental impact, and improved scalability due to reliance on renewable and biodegradable natural resources [23, 24]. Moreover, research has shown that incorporating AgNPs into plant extracts increases the concentration of flavonoids and phenolic compounds, leading to enhanced antioxidative and antibacterial efficacy compared to both the raw extract and AgNO_3_ alone [25]. Additionally, pharmaceuticals can be incorporated into nanomaterials, allowing for targeted organ delivery without causing significant side effects [26].

This study aims to investigate the potential of *Moringa oleifera* loaded with silver nanoparticles (MO-AgNPs) as a novel anti-obesity therapy. This combination not only enhances the therapeutic efficacy of Moringa’s bioactive compounds but also introduces the unique properties of AgNPs, such as their ability to modulate critical metabolic and thermogenic pathways. Specifically, the expression of genes like UCP1 and PPARGC1A; measurement of these expressions in blood gives us a real picture about these values, which are involved in energy expenditure and lipid metabolism, can be upregulated, providing a new approach to combat obesity and its related complications. By integrating the natural anti-obesity effects of Moringa with the enhanced functionality of AgNPs, this study investigates the potential of MO-AgNPs as an effective, innovative, and less toxic treatment option for obesity management. So, our hypothesis in this study is that *Moringa oleifera* specially in its new formula (MO-AgNPs) is being researched as a potent hypolipidemic agent and could play a beneficial role in management of obesity and improvement of its complications. Thus, the aim of this study was to explore the impact of genetic variations in PPARGC1A and UCP1 on obesity and evaluate the hypolipidemic efficacy of AgNP-encapsulated *Moringa oleifera* extract.

## Methods

### Drugs and Reagents

All chemicals used in this study were obtained from Sigma-Aldrich Corporation, St. Louis, Missouri, USA. *Moringa oleifera* leaves were harvested from mature *Moringa* trees cultivated in the Assiut Governorate, Upper Egypt, a region characterized by a hot arid climate and well-drained sandy-loam soil, which are favorable for the optimal growth and phytochemical accumulation in *Moringa* plants. This specific geographic origin was selected due to its consistent agro-ecological conditions that enhance the biosynthesis of bioactive compounds. The plant material was taxonomically identified and authenticated by a botanist at the Faculty of Science, Assiut University, and a voucher specimen (MO-2025-EG) was deposited in the departmental herbarium.

### Preparation of Alcoholic Extract of *Moringa Oleifera*

*Moringa oleifera* leaves were ground into powder using a blender. Fatty materials were initially extracted by macerating the leaves in petroleum ether. The resulting marc was then extracted three times with 5 L of 70% ethanol over a period of 3 days. The extract was further immersed in ethyl alcohol for 7 days, with daily agitation, and then filtered using cotton swabs. The filtered extract was concentrated using a rotary evaporator (Buchi, USA). The extraction process was repeated under reduced pressure until a clear supernatant was obtained, which was subsequently lyophilized (Labconco, USA). The extract was stored in an airtight container at a temperature lower than 10 °C for further use.

A 70% hydroethanolic solvent was selected to ensure comprehensive extraction of both water-soluble and ethanol-soluble bioactive compounds, which are believed to contribute synergistically to the plant’s therapeutic properties.

### High-Performance Liquid Chromatography (HPLC)-Assay of Flavonoids and Phenolic Compounds in *Moringa Oleifera* Extract

Agilent Technologies 1100 series liquid chromatography was used for HPLC analysis of phenolic and flavonoid compounds. which is equipped with an auto sampler and a diode-array detector. Phenomenex, Torrance, CA, supplied the Eclipse XDB-C18 (150 × 4.6 μm; 5 μm) analytical column, which was outfitted with a C18 guard column. Acetonitrile (solvent A) and 2% acetic acid in water (v/v) (solvent B) made up the mobile phase. The steps in the gradient program were as follows: in 30 min, go from 100% B to 85% B; in 20 min, go from 85% B to 50% B; in 5 min, go from 50% B to 0% B; and in 5 min, go from 0% B to 100% B. The 70-minute run was conducted with the flow rate maintained at 0.8 ml/min. The derivatives of benzoic acid and cinnamic acid were found to have simultaneous peak detection at 280 and 320 nm, respectively, with an injection volume of 50 µl. Prior to injection, each sample was passed through an Acrodisc syringe filter (Gelman Laboratory, MI) with a 0.45 μm particle size. Congruent retention periods and UV spectra were used to locate the peaks, and their values were compared to the standards [12].

### Synthesis of AgNPs-Encapsulated *Moringa Oleifera* (MO-AgNPs)

In a 250 mL flask, 10 mL of *Moringa oleifera* (MO) extract was mixed with 90 mL of a freshly prepared 1 mM silver nitrate aqueous solution (AgNO_3_) and constantly stirred using a hot plate magnetic stirrer at a rotation speed of 200 rpm at 60 °C under dark conditions. After 30 min, the mixture solution became turbid and changed from yellow to reddish-brown, indicating the formation of silver nanoparticles (AgNPs). To purify the AgNPs and remove residual MO extract, the suspension was centrifuged three times at 15,000 rpm for 20 min to collect the dark brown precipitate. The precipitate was washed twice with double-sterilized water and once with methanol to ensure the removal of unreacted components. Finally, the powder precipitate was dried to yield AgNPs. To optimize the production conditions for AgNPs using MO extract, various experimental factors were varied, such as the concentration of MO extract, the contact time, and the concentration of AgNO_3_.

### Statistical Optimization of MO-AgNPs Synthesis

Response Surface Methodology (RSM) is a powerful mathematical and statistical tool used to model processes efficiently while minimizing the number of experiments required. It offers valuable insights into the effects of multiple variables and their interactions, enabling the identification of optimal conditions for desired outcomes. In this study, a 3² full factorial design was employed using Design Expert^®^ version 13.0.5 software (Stat-Ease, Inc., Minneapolis, Minnesota, USA) to assess the impact of three independent variables: the extract-to-AgNO_3_ ratio (X₁), pH (X₂), and temperature (X₃). Each factor was evaluated at two distinct levels, as presented in Table 1. The evaluation was done for three responses: particle size (PS, Y₁), polydispersity index (PDI, Y₂), and zeta potential (ZP, Y₃). Preliminary experiments helped define appropriate ranges for each factor to ensure meaningful and reliable results. All experiments were conducted in triplicate to reduce variability and enhance data accuracy. The experimental design matrix, including both coded and actual levels of the factors, is provided in Table 2. The data was analyzed using Analysis of Variance (ANOVA) to determine the statistical significance of individual factors and their interactions. Polynomial regression equations were developed to predict the responses under varying experimental conditions, guiding the optimization of synthesis parameters for producing nanoparticles with minimal particle size, narrow size distribution, and maximum zeta potential.

**Table 1:** Factors and responses with their respective constraints of the full factorial design for green synthesis of silver nanoparticles using *Moringa Oleifera* leaves

Independent Variables	Levels	
	Low	High
X_1_: Extract to AgNO3 Ratio (%v/v)	10:90	20:80
X_2_: pH	7	10
X_3_: Temperature (ºC)	35	70
Dependent Variables	**Desirability Constraints**	
Y_1_: PS (nm)	Minimize	
Y_2_: PDI	Minimize	
Y_3_: ZP (-mV)	Maximize	

*PS* particle size, *PDI* polydispersity index, *ZP* zeta potential

**Table 2:** (i) Full factorial design matrix for the optimization of MO-AgNPs synthesis and the observed responses; (ii) summary of the regression analysis and model statistics

i)	Independent Variables			Dependent Variables		
	**X**_**1**_: **Extract to AgNO**_**3**_ **Ratio**	**X**_**2**_: **pH**	**X**_**3**_: **Temperature**	**Y**_**1**_: **PS**	**Y**_**2**_: **PDI**	**Y**_**3**_: **ZP**
	(%v/v)		(ºC)	(nm)		(-mV)
M1	10:90	10	35	32.95 ± 8.68	0.31 ± 0.02	24.56 ± 1.05
M2	10:90	10	70	29.81 ± 5.14	0.29 ± 0.05	25.71 ± 0.09
M3	20:80	10	70	25.74 ± 4.65	0.21 ± 0.04	30.45 ± 1.56
M4	10:90	7	35	40.04 ± 10.23	0.43 ± 0.10	20.02 ± 0.87
M5	20:80	7	35	34.92 ± 6.36	0.35 ± 0.05	25.75 ± 0.56
M6	20:80	7	70	31.63 ± 5.58	0.3 ± 0.06	27.94 ± 0.89
M7	10:90	7	70	35.45 ± 5.76	0.38 ± 0.02	21.54 ± 0.97
M8	20:80	10	35	28.16 ± 4.54	0.24 ± 0.08	28.46 ± 1.01
ii)						
R ^2^				0.9997	0.9997	0.9999
Adjusted R ^2^				0.9980	0.9976	0.9997
Predicted R ^2^				0.9813	0.9780	0.9973
Adequate precision				73.7943	65.7658	184.7580
Significant factors and interactions				X_1_, X_2_, X_3_	X_1_, X_2_, X_3_	X_1_, X_2_, X_3,_ X_1_ × _2_

### Bio-Green Synthesis of Silver Nanoparticles Using Soluble Starch

AgNPs were synthesized using a nanoprecipitation method for the biochemical comparison with MO-AgNPs. First, 1.5 g of soluble starch was dissolved in 35 mL of deionized water with 0.01 g of sodium hydroxide (NaOH) to adjust the pH. The mixture was heated to 60 °C and stirred until the complete dissolution of the starch. Separately, 0.5 g of silver nitrate (AgNO₃) was dissolved in 10 mL of deionized water. To the starch solution, 0.5 g of Tween 80 was added dropwise while stirring. The silver nitrate solution was then gradually added to the starch-Tween 80 mixture, and the reaction was stirred at 60 °C for 30 min. The solution turned reddish-brown, confirming the synthesis of AgNPs. The nanoparticles were purified by centrifugation at 15,000 rpm for 20 min and washed with double-sterilized water and methanol before being dried [27].

### Characterization of the Synthesized AgNPs

#### UV-Visible Spectral Analysis

A UV-visible spectrophotometer was used to monitor the formation of AgNPs by measuring the absorbance of the reaction mixture over the wavelength range of 400–450 nm, corresponding to the surface plasmon resonance (SPR) of AgNPs. The appearance of an SPR peak confirmed the successful synthesis of AgNPs.

#### Transmission Electron Microscopy (TEM)

The chosen Ag-NPs formulation’s morphology and particle size were assessed through transmission electron microscopy (TEM) (JEOLJEM1230, Tokyo, Japan). A drop of diluted sample was examined by staining it with 2% (w/v) phosphotungestic acid on a copper grid. The micrograph was obtained at an appropriate magnification power, and the experiment was carried out at room temperature.

### Animals

Fifty male albino rats weighing between 180 and 200 g purchased from Egyptian Organization for Biological Products and Vaccines (Cairo, Egypt) were recruited for the present study at the beginning of the experiment. Every rat was housed in a hygienic polypropylene cage with a 12-hour cycle of light and dark to maintain a controlled temperature of 22 ± 2 °C. Forty male albino rats were given a Western-style diet which known as the high-carbohydrate; high-fat (HCHF) diet (WD; 21.2% fat, 34% sucrose, and 0.2% cholesterol by weight) and the other ten rats were given a regular chow diet as the control diet (CD; 5.2% fat, 12% sucrose, and 0.01% cholesterol by weight) [28]. In this case, it is utilized to build a model that more accurately depicts how obesity develops in people. Cooking methods were customary [29]. The animals were allowed fourteen days before the trial to get used to the laboratory environment. Animals were anesthetized using light ether prior to cervical dislocation. Formalin was used postmortem only for fixation. The National Institutes of Health’s recommendations for the use and care of laboratory animals, along with ethical guidelines and the approval of the Faculty of Pharmacy, Ahram Canadian University ethical committee under the reference number REC2124, were adhered to during all procedures.

#### Experimental Design

Out of fifty male albino rats, five groups of 10 were chosen at random. Group I (Control): Healthy rats were fed a standard diet. Group II (HCHFD): A high-carb, high-fat diet was given to the rats for 20 weeks. Prior to starting oral AgNPs treatment (10 mg/kg bw/day), rats in Group III (AgNPs + HCHFD) were fed a diet heavy in fat and carbohydrates [26]. Before beginning an 8-week treatment of 400 mg/kg body weight of *Moringa oleifera* extract, rats in Group IV (MO + HCHFD) were fed a diet heavy in fat and carbohydrates [9]. Group V (MO-AgNPs + HCHFD): A high-carb, high-fat diet was given to the rats for eight weeks prior to receiving an oral dose of MO-AgNPs (10 mg/kg bw/day).

The dose of *Moringa oleifera* (400 mg/kg) was selected based on previous studies demonstrating its anti-obesity efficacy and safety in vivo. For example, methanolic extracts of *Moringa oleifera* leaves administered at 200 mg/kg and 400 mg/kg to high-fat diet–induced obese rats produced significant reductions in body weight, serum cholesterol, triglycerides, LDL, and liver biomarkers, while enhancing HDL and thermogenic activity without affecting feed intake [30]. The dose of 400 mg/kg was reported to exert stronger hypolipidemic and thermogenic effects, which guided our selection for this study.

Recent research has shown that MO-AgNPs are biocompatible and protective in vivo. The hematological effects of MO-AgNPs in male albino rats exposed to acrylamide-induced toxicity were examined by El-Bakry et al. (2022). The study found that acrylamide-induced hematological changes, such as the normalization of hemoglobin levels, red and white blood cell counts, and other blood parameters, were considerably reduced by oral administration of MO-AgNPs (50 mg/kg body weight). These results support the safety of MO-AgNPs for use in biomedical applications by indicating that they have protective qualities and do not cause adverse hematological toxicity at the evaluated dose [31].

For AgNPs, the dose of 10 mg/kg was chosen based on earlier reports showing efficacy and safety in obesity models. In a study evaluating diet-induced obesity in rats, AgNPs administered at 10 mg/kg and 20 mg/kg significantly reduced adipocyte size, alleviated hepatic steatosis, improved oxidative stress markers, and lowered pro-inflammatory cytokines [20]. The therapeutic effects observed at 10 mg/kg, combined with a favorable safety profile, supported our selection of this dose.

### Collection of Samples

The animals were fast for eight hours following the 28-week study before their blood was drawn. After giving the individuals, a formalin injection to put them to sleep, using capillary tubes, blood was extracted from the eye’s retroorbital venous plexus, collected in clean tubes, allowed to clot, and centrifuged for ten minutes at 3000 r.p.m. The serum was separated and stored in an Eppendorf container at −20 °C to ascertain its biochemical properties after the blood samples were extracted, then kept at −80 °C until they were needed for studies on gene expression. Rats were sacrificed by dislocating their cervical vertebrae, and their pancreas and livers were stored in 10% formalin phosphate buffer for further histological analysis.

### Biochemical Analysis

#### Determination of Glucose Level

The typical commercial enzymatic colorimetric assays (Roche Diagnostics, Basel, Switzerland; BioMerieux, Marcy l’Etoile, France) were employed to determine the serum glucose level in accordance with the protocols [32].

#### Determination of Insulin Level

The serum insulin level was measured using an enzyme-linked immunological sorbent assay kit from BioSoure INSEASIA Co. (Nivelles, Belgium) [33]. Insulin resistance was calculated using the homeostatic model assessment for insulin resistance, or HOMA-IR formula, is equivalent to fasting glucose (mg/dl) × fasting insulin (µIU/ml)/405 [34].

The new quantitative insulin sensitivity check index (R-QUICKI) is calculated as follows: 1/[log fasting glucose (mg/dl) + log fasting insulin (µIU/ml)]. was employed to assess sensitivity to insulin.

#### Determination of Lipid Profile Level

The application of colorimetric enzymatic assays that are industry standard (BioMerieux, France; Roche Diagnostics, Basel, Switzerland), serum levels of cholesterol [35], triglycerides (TG) [36], and high-density lipoprotein (HDL-cholesterol) [37]. The following formula was used to calculate LDL-cholesterol: LDL-C (mg/dl) = Total cholesterol - (HDL-C + TG/5) [38].

#### Determination of Serum Levels of Leptin, Adiponectin, Resistin, and Irisin

Leptin (Catalog Number KRC2281, Thermo Fisher Scientific Inc., USA), Adiponectin (Rat Adiponectin ELISA Kit PicoKine^®^ (Boster Biological Technology, Pleasanton CA, USA, Catalog # EK1327), resistin (Catalog number ab289699, Abcam, United Kingdom) and irisin (Catalog Number orb567771, Biorbyt, United Kingdom) levels were determined using Enzyme-linked immunosorbent assay (ELISA) employing the manufacturer’s protocols for rat (R&D systems).

#### Expression of UCP1 and PPARGC1A Genes by Reverse Transcription-Quantitative Polymerase Chain Reaction (RT-qPCR)

The RNase Kit (Qiagen, Germany) was used to extract total RNA from blood samples. Using a spectrophotometer with an acceptable A260/A280 ratio of 1.8 to 2.1, the RNA’s purity and concentration were confirmed by analyzing at 260 and 280 nm optical density (OD).

Following the manufacturer’s instructions, the extracted RNA underwent reverse transcription. In a 1.5-hour incubation at 42 °C, 1 µg of total RNA, 25 nanomoles of dNTPs (Promega), 1 µg of polyT primers, and 5 µl of M-MLV reaction buffer (Promega) were mixed with 200 units of M-MLV-RTase (Promega). 5 µl of the RT reaction were added to 25 µl of a mixture that included 1 micrometer each of forward and reverse primers, 2.5 units of Eurobio Taq DNA polymerase, 12.5 µl of 2x QuantiFast SYBR Green PCRMaster Mix, and 1.5 millimolar MgCl2. The internal reference gene used was β-actin (Table 3). PCR was subsequently carried out.

**Table 3:** β-actin, UCP1, and PPARGC1A gene primer sequences

Target	Sequence
β-actin	F: 5′-AGGGAAATCGTGCGTGACAT-3′ R: 5′-GAACCGCTCATTGCCGATAG-3′
UCP1	F: 5′-GTGAAGGTCAGAATGCAAGC-3′ R: 5′-AGGGCCCCCTTCATGAGGTC-3′
PPARGC1A	F: 5′-GCT TGA CTG GCG TCA TTC A-3′ R: 5′-ACA GAG TCT TGG CTG CAC ATG T-3′

The thermal profile comprised 45 cycles of denaturation at 95 °C for 15 s, annealing (56–60 °C) for 30 s, and extension at 72 °C for 45 s. The initial denaturation took place at 95 °C for 5 min. The fluorescence of amplified genes was measured using the SyberGreen Master Mix. Using melting curve analysis, the amplification’s specificity was tracked. Measurement of the fold change in mRNA expression was done using **Livak and Schmittgen** method [39]. Taqman PCR assays and reagents from Applied Biosystems and QuantStudio 12 K Flex real-time PCR assays were used to determine the expression level.

### Histopathological Examination

After the experimental group’s liver and pancreas samples were removed, they were fixed in a buffered formalin solution for forty-eight hours. Following this, they were rinsed with distilled water, soaked in an escalating alcohol series, extracted using xylene, and then imbedded in paraffin wax. The tissue was initially sectioned into 5 μm sections, stained with hematoxylin and eosin, and subsequently mounted in DPX. We used a light microscope to look for histopathological problems in the stained sections.

### Statistical Analysis

The information was provided as mean values and standard errors of the mean (SEM). The data proved to be a normal distribution with the help of a normality test (SPSS software, version 26). For trials with more than two groups and one variable, we used one-way analysis of variance (ANOVA) followed by post hoc Bonferroni testing to determine statistical significance. Pearson correlation coefficient could be calculated. Statistical significance (P) was defined as a probability value that is less than 0.05.

## Results and Discussion

### High-Performance Liquid Chromatography (HPLC) Apparatus Analyzing *Moringa Oleifera* Extract for Phenolic and Flavonoid Chemicals

Our previous study showed that phenolic compounds, specifically p-hydroxybenzoic acid (2673.59 µg/g), sinapic acid (332.60 µg/g), caffeic acid (86.53 µg/g), gallic acid (77.16 µg/g), p-coumaric acid (59.19 µg/g), ferulic acid (56.39 µg/g), chlorogenic acid (48.56 µg/g), as well as flavonoids, including catechin (2045.44 µg/g), rutin (599.05 µg/g), quercetin (245.95 µg/g), and cinnamic acid (57.96 µg/g) [40].

### Statistical Analysis of the Full Factorial Design

**Fig. 1:**
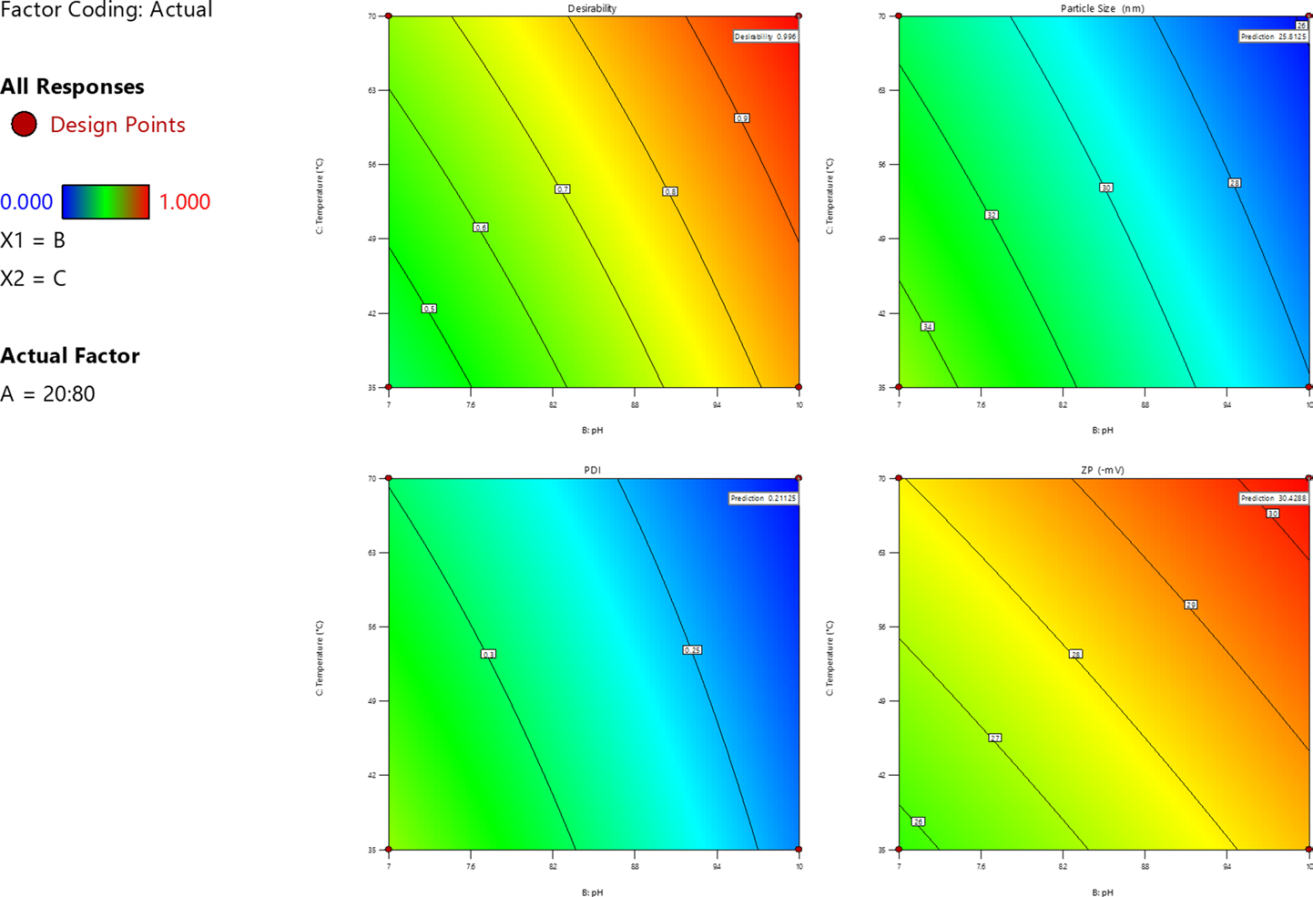
Contour plots for the effects of extract-to-AgNO3 ratio (X₁), pH (X₂), and temperature (X₃). on particle size (PS, Y₁), polydispersity index (PDI, Y₂), and zeta potential (ZP, Y₃) of green synthesis of silver nanoparticles using *Moringa Oleifera* leaves

A 2^3^ full factorial design was utilized using Design Expert^®^ version 13.0.5 software to evaluate the effects of three independent variables on the properties of CS-ZnO nanoparticles (NPs). The factors included the extract-to-AgNO₃ ratio (X₁), pH (X₂), and temperature (X₃). The measured responses were particle size (PS), polydispersity index (PDI), and zeta potential (ZP) (Table 1). ANOVA tests were employed to assess the significance of the factors in each response. High R^2^ values signified a strong correlation between predicted and experimental values. Adequate precision values (signal-to-noise ratio > 4) confirmed the reliability of the model, and a non-significant lack of fit supported its validity. The Box-Cox plots indicated no need for data transformation. The adjusted and predicted R2 were in reasonable agreement for all the dependent variables and were described in Table 2(ii). The contour plots (Fig. 1) visualized the influence of significant variables on each response, providing insights into optimal conditions and predicted values. Predictive models expressed in coded factors were used to analyze the main effects, as well as two-factor and three-factor interactions, based on the equations:

1 $${\mathrm Y}_1=32.338-2.225\;{\mathrm X}_1-3.173\;{\mathrm X}_2-1.680\;{\mathrm X}_3+0.010\;{\mathrm X}_1{\mathrm X}_2+0.252\;\;{\mathrm X}_1{\mathrm X}_3+0.290\;{\mathrm X}_2{\mathrm X}_3$$

2 $${\mathrm Y}_2=0.314-0.039\;{\mathrm X}_1-0.051\;{\mathrm X}_2-0.019\;{\mathrm X}_3+0.001\;{\mathrm X}_1{\mathrm X}_2-0.001\;{\mathrm X}_1{\mathrm X}_3+0.006\;\;{\mathrm X}_2{\mathrm X}_3$$

3 $${\mathrm Y}_3=25.554+2.596\;{\mathrm X}_1+1.741\;{\mathrm X}_2+0.856\;{\mathrm X}_3-0.436\;{\mathrm X}_1{\mathrm X}_2+0.189\;{\mathrm X}_1{\mathrm X}_3-0.071\;\;{\mathrm X}_2{\mathrm X}_3$$

Positive coefficients indicate synergistic effects, while negative coefficients reflect antagonistic effects. Larger coefficients signify a stronger influence of the corresponding variable on the response.

Equation (1) illustrates the effect of the relative proportion of the three studied variables on particle size response (Y1). The extract-to-AgNO_3_ ratio (20:80) produced smaller particle sizes due to the higher concentration of MO extract, which enhances nanoparticle capping and stabilization, thereby preventing excessive growth. It has been observed that increasing the pH value leads to a reduction in nanoparticle size, enhanced absorption, and more uniform size distribution. In an alkaline medium, nanoparticles exhibit improved stability and reduced tendency for agglomeration, promoting the formation of stable colloids [41]. Alkaline conditions also offer additional advantages, such as a stable and high yield of nanoparticles, accelerated growth rate, and an improved reduction process [42]. The role of OH groups in plant extracts is critical in the synthesis process, acting as both reducing and stabilizing functional groups. A basic pH facilitates the involvement of more OH groups in the reduction reaction, which improves the reduction yield and contributes to the stability of AgNPs [43]. This aligns with Sathishkumar et al. theory, which suggests that at higher pH values, the abundance of functional groups promotes rapid binding with Ag(I) ions, resulting in smaller and more uniformly sized nanoparticles [44].

Temperature factor (X3) played a critical role, with a synthesis temperature of 70 °C yielding smaller nanoparticles compared to 35 °C. This may be attributed to accelerated reaction kinetics and faster nucleation processes that restrict particle growth. Studies have shown that as the reaction temperature increases, the size of AgNPs decreases, accompanied by changes in their morphology [45, 46]. Elevated temperatures facilitate the rapid reduction of Ag⁺ ions and subsequent nucleation, leading to the formation of smaller, spherical nanoparticles. Conversely, lower reaction temperatures promote nanoparticle growth, resulting in larger particle sizes [47]. Efficient synthesis of AgNPs has been reported at temperatures between 60 and 80 °C, demonstrating the importance of maintaining an optimal temperature range for achieving desirable nanoparticle properties [48].

Equation (2) demonstrates that PDI values were lower at the higher extract-to-AgNO_3_ ratio, reflecting a more uniform size distribution. Conversely, the 10:90 ratio led to broader distributions due to inadequate stabilization. PDI values were also smaller at pH 10, indicating better nanoparticle size control. Higher temperatures (70 °C) yielded slightly lower PDI values, suggesting reduced aggregation due to rapid nucleation.

Equation (3) highlights the effect of the relative proportion of the three studied variables on ZP response (Y3). Zeta potential measurements also indicated improved electrostatic stability with the at the higher level of extract-to-AgNO₃ ratio, as the higher MO concentration ensures better surface stabilization. Additionally, zeta potential values were more negative at pH 10, signifying superior electrostatic stabilization compared to pH 7. Marginal improvements in zeta potential were observed at 70 °C, likely due to stronger interactions between MO extract and nanoparticles during synthesis.

Using statistical experimental designs and the desirability function, the optimal system was determined by targeting minimal PS and PDI values while maximizing ZP. The software identified the M3 formulation, which utilized an extract-to-AgNO₃ ratio of 20:80, a pH of 10, and a temperature of 70 °C, achieving an overall desirability score of 0.996. Therefore, M6 was selected for further investigations and biochemical studies.

### UV-Visible Spectral Analysis

**Fig. 2:**
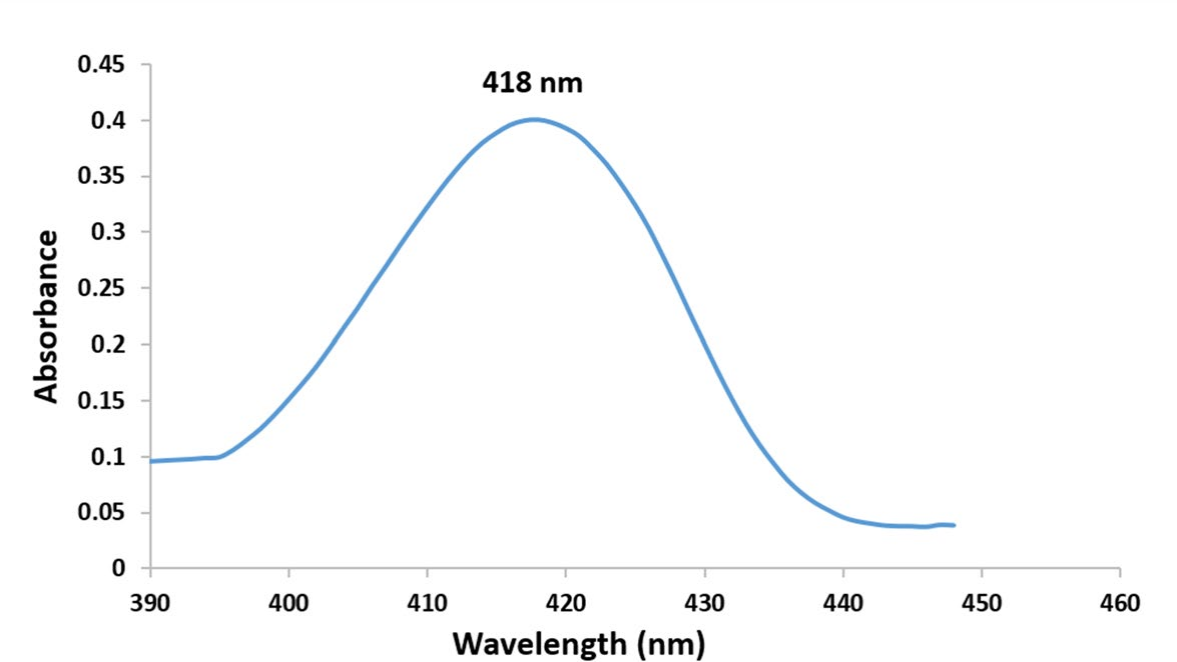
UV-visible spectroscopy of green synthesized silver nanoparticles using *Moringa Oleifera* leaves extract

The optical properties of the synthesized AgNPs were analyzed using UV-Visible spectroscopy. Figure 2 displays the UV-Vis absorption spectrum of the AgNPs dispersed in deionized water. The spectrum shows a characteristic surface plasmon resonance (SPR) absorption peak in the range of 400–450 nm, with the maximum absorbance observed at approximately 418 nm. This peak is attributed to the collective oscillation of conduction band electrons in the AgNPs induced by interaction with light. The position of the absorption peak aligns with previously reported values in the literature for AgNPs synthesized via green chemistry approaches [49, 50].

### Transmission Electron Microscopy (TEM)

**Fig. 3:**
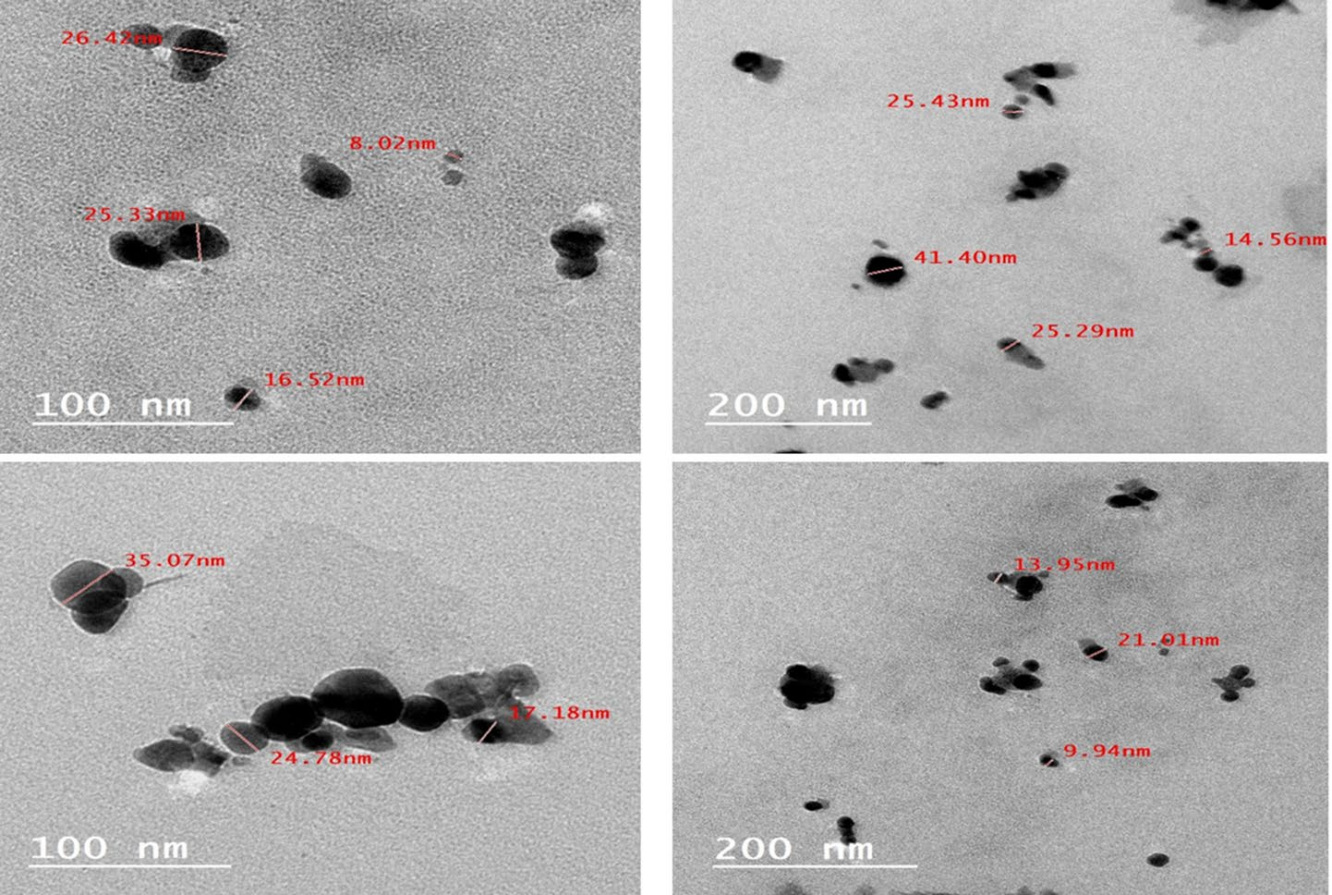
TEM of *Moringa Oleifera* -loaded silver nanoparticle (MO-AgNPs)

Transmission Electron Microscopy (TEM) is a valuable tool for analyzing the shape and morphology of AgNPs. TEM examination of the synthesized AgNPs revealed that the nanoparticles were primarily spherical with a uniform appearance (Fig. 3). This observation aligns with findings at higher pH and temperature levels, where nanoparticles tend to exhibit spherical shapes, and the reaction rate is notably accelerated [51]. The spherical morphology suggests that biomolecules from the extract effectively acted as capping and stabilizing agents during synthesis, playing a dual role as reducing and stabilizing agents. Dynamic Light Scattering (DLS) analysis demonstrated a mean hydrodynamic diameter of 25.74 ± 10.14 nm, which closely corresponds with the sizes observed in TEM images. The consistency between DLS and TEM data highlights the accuracy and reliability of the particle size characterization.

### Biochemical Analysis

The excessive buildup of adipose tissue brought on by a persistent imbalance between caloric intake and energy expenditure is what defines obesity. Numerous metabolic disorders, such as dyslipidemia, hyperglycemia, poor glucose tolerance, and hormonal dysregulation, are intimately linked to it. Along with these issues, obesity raises the risk of many chronic diseases and is a contributing factor to higher rates of morbidity and death. Achieving safe and long-lasting weight loss is still difficult, even with continuous improvements in pharmaceutical treatments, because many procedures have unfavorable side effects [52]. Consequently, the current study’ objective was to investigate the impacts of combining silver nanoparticles loaded with *Moringa Oleifera* in the treatment of metabolic problems caused by obesity.

Silver, a vital metallic element, plays a crucial role in various metabolic processes, including glucose metabolism. Despite its importance, limited research has been conducted to explore the impact of silver or silver nanoparticles on glucose regulation. Studies suggest that metals are significantly involved in glucose metabolism, and deficiencies in certain metals have been associated with complications in individuals with diabetes. In this study, we utilized the reducibility and stability properties of soluble starch along with tween 80, an environmentally friendly surfactant, to prevent agglomeration and ensure effective dispersion of the resulting nanocomposite [26].

The findings in Table 4 demonstrate that fasting blood sugar and HOMA-IR levels were significantly lower in all treatment groups compared to the HCHFD group. In contrast, insulin levels and R-QUICKI scores were significantly higher in the treatment groups, indicating improved insulin sensitivity and glucose metabolism. The group treated with MO + HCHFD showed a significant reduction in glucose levels and HOMA-IR, along with increased insulin levels and R-QUICKI scores when compared to the AgNPs + HCHFD group. Similarly, the MO-AgNPs + HCHFD group exhibited significant differences in insulin levels, HOMA-IR, and R-QUICKI scores compared to the AgNPs + HCHFD group. However, no substantial differences were observed between the MO-AgNPs + HCHFD and MO + HCHFD groups across these parameters (*P* < 0.05).

**Table 4:** Fasting glucose, insulin, HOMA-IR and R-QUICKI in different groups

	Control *n* = 10	HCHFD *n* = 10	AgNPs + HCHFD *n* = 10	MO + HCHFD *n* = 10	MO-AgNPs + HCHFD *n* = 10
Glucose (mg/dl)	73.65 ± 1.31	175.67 ± 2.23 ^a^	80.92 ± 1.87 ^b^	91.97 ± 1.66 ^abc^	77.23 ± 2.24 ^bd^
Insulin (µIU/ml)	12.06 ± 0.43	6.36 ± 0.17 ^a^	8.28 ± 0.15 ^ab^	9.97 ± 0.15 ^abc^	10.8 ± 0.23 ^abc^
HOMA-IR	2.19 ± 0.09	2.76 ± 0.07 ^a^	1.66 ± 0.05 ^ab^	2.26 ± 0.04 ^bc^	2.06 ± 0.07 ^bc^
R-QUICKI	0.34 ± 0.002	0.33 ± 0.001 ^a^	0.35 ± 0.002 ^ab^	0.34 ± 0.001 ^bc^	0.34 ± 0.002 ^bc^

Mean SE is the statistical distribution used to represent values. For each group, n is the total number of rats. A p-value < 0.05 was interpreted as demonstrating statistical significance

a significant when compared to control group at *P* < 0.05

b significant when compared to HCHFD group at *P* < 0.05

c significant when compared to AgNPs + HCHFD group at *P* < 0.05

d significant when compared to MO + HCHFD group at *P* < 0.05

According to Imran et al. [53], our results are supported by the fact that nanomaterials with unique qualities including high surface area, small size, improved solubility, and biocompatibility are employed as antidiabetic medicines. In comparison to the crude medication, the NPs small size enables them to exist as transported past the cell membrane by a transport mechanism, which could be the cause of any possible biological effects.

The existence of phenolic chemicals, which are strong antioxidants that may scavenge reactive oxygen species, further supports this. It’s probable that the flavonoids and phenols in MO scavenged the reactive oxygen species (ROS) that mitochondria created, safeguarding beta cells and reversing hyperglycemia, as proposed by Al-Malki and El Rabey [54]. Alkhalidy et al. [55] found that Mo therapy enhances glucose absorption, skeletal muscle glycolysis, glycogen synthesis, AMPK activity, and GLUT-4 expression.

The results in Table 5 showed a substantial increase in HDL-cholesterol levels and a substantial decrease in LDL-cholesterol, cholesterol, and triglyceride levels in all treatment groups compared to the HCHFD group. Moreover, there was no discernible change between the groups treated with AgNPs and MO-AgNPs; on the other hand, a significant difference was noted between the groups treated with MO alone and MO-AgNPs. The groups received silver nanoparticles and *Moringa* showed an improvement in lipids profile compared to HCHFD group while the group which received the mixture from silver nanoparticle and *Moringa* showed remarkable progress in lipids profile at *P* < 0.05. One of the main indicators of the pathophysiology of obesity is hyperlipidemia, which is defined as elevated levels of HDL and triglyceridemia and cholesterol respectively. One major risk factor for cardiovascular conditions like atherosclerosis is thought to be hyperlipidemia [9]. When comparing all treated groups to the HCHFD group, our findings in Table 5 revealed a substantial rise in HDL-cholesterol and a substantial reduction in triglycerides, cholesterol, and LDL-cholesterol. Furthermore, there was no discernible difference between the groups treated with AgNPs and MO-AgNPs; on the other hand, a significant difference was noted between the groups treated with MO alone and MO-AgNPs. The lipid profiles of the groups that got silver nanoparticles and *Moringa* improved, whereas the group that received the mixture of silver nanoparticles and *Moringa* had a significant improvement in lipid profiles at *P* < 0.05.

**Table 5:** Lipids profile in different groups

	Control *n* = 10	HCHFD *n* = 10	AgNPs + HCHFD *n* = 10	MO + HCHFD *n* = 10	MO-AgNPs + HCHFD *n* = 10
Cholesterol (mg/dl)	109.63 ± 1.33	169.58 ± 2.85 ^a^	139.00 ± 1.06 ^ab^	143.97 ± 1.35 ^ab^	109.27 ± 1.7 ^bcd^
Triglyceride (mg/dl)	75.96 ± 0.74	161.84 ± 1.09 ^a^	134.06 ± 0.74 ^ab^	144.75 ± 0.89 ^abc^	76.24 ± 1.06 ^bcd^
HDL-cholesterol (mg/dl)	71.1 ± 1.2	32.38 ± 0.61 ^a^	42.49 ± 1.03 ^ab^	39.73 ± 0.69 ^ab^	68.98 ± 1.49 ^bcd^
LDL-cholesterol (mg/dl)	23.33 ± 1.36	104.83 ± 2.85 ^a^	74.67 ± 1.54 ^ab^	75.29 ± 1.91 ^ab^	25.04 ± 1.83 ^bcd^

Mean SE is the statistical distribution used to represent values. For each group, n is the total number of rats. A p-value < 0.05 was interpreted as demonstrating statistical significance

a significant when compared to control group at *P* < 0.05

b significant when compared to HCHFD group at *P* < 0.05

c significant when compared to AgNPs + HCHFD group at *P* < 0.05

d significant when compared to MO + HCHFD group at *P* < 0.05

*Moringa olifera’s* antihyperlipidemic and hypoglycemic effects against obesity may be due to phytochemicals such phenolic, flavonoids, b-sitosterol, and saponins, which are contained in this plant extract and have important functions in lipid regulation. They are phytochemicals that significantly slow and decrease the absorption of carbohydrates. Moreover, they may improve bile acid binding, which can lower plasma cholesterol levels by forming insoluble complexes and increasing excretion in the stool [56, 57]. Additionally, by binding to bile acids and decreasing their enterohepatic circulation, the saponins in *Moringa olifera* can prevent cholesterol absorption. This also increases the amount of cholesterol expelled in the feces, which decreases plasma cholesterol and lessens hyperglycemia and insulin resistance [58].

Since MO is an antioxidant, the general mechanism by which the MO extract can improve the symptoms of metabolic syndrome is believed to exist. According to Yassa and Tohamy [59], MO is considered a rich reservoir of metabolites with antioxidant potential that may have an impact on cholesterol and blood sugar levels. Because of this, the extract’s phenolic components play a significant part in its hypolipidaemic effects. For example, it has been noted that Quercetin-O-rhamnosylhexosyl (rutin), one of the primary phenols in our MO extract, lowers serum total lipids, cholesterol, TGs, LDL-cholesterol, and VLDL-cholesterol in diabetic rats. Additionally, quercetin aglycone which was also extracted in MO extract along with its other glycosides showed antihyperlipidaemic properties in treated rats [9].

Additionally, the delivery of silver nanoparticles to rats resulted in a significant (*P* < 0.05) decrease in the serum lipid profile. This fall was caused by the AgNPs entering bloodstream and being removed by macrophages, the first cell to interact with cholesterol and NP via a scavenger receptor. Consequently, the uptake and apoptosis are facilitated by the contact between AgNP and the receptor on the surface of macrophages. The scavenger receptor interacts with the lipid metabolism rate to accelerate the development of atherosclerosis [60].

The treatment groups showed statistically significant decreases in levels of leptin and resistin, along with increases in adiponectin and irisin, compared to the HCHFD group (Table 6). Among the treated groups, the combination of silver nanoparticles loaded with *Moringa oleifera* (MO-AgNPs) demonstrated the most remarkable improvement, with levels of leptin, adiponectin, resistin, and irisin nearly matching those of the control group (*P* < 0.05). This suggests that the MO-AgNPs treatment effectively restored adipokine balance, alleviating the metabolic disturbances associated with obesity.

**Table 6:** Serum leptin, adiponectin, resistin and irisin in different groups

	Control *n* = 10	HCHFD *n* = 10	AgNPs + HCHFD *n* = 10	MO + HCHFD *n* = 10	MO-AgNPs + HCHFD *n* = 10
Leptin (pg/ml)	2.5 ± 0.09	4.32 ± 0.11 ^a^	3.08 ± 0.08 ^ab^	3.6 ± 0.07 ^abc^	2.76 ± 0.07 ^bd^
Adiponectin (ng/ml)	11.46 ± 0.54	6.36 ± 0.17 ^a^	9.19 ± 0.12 ^ab^	7.73 ± 0.21 ^abc^	9.59 ± 0.2 ^abd^
Resistin (pg/ml)	32.9 ± 0.53	50.85 ± 0.72 ^a^	39.33 ± 1.01 ^ab^	40.4 ± 0.67 ^ab^	37.46 ± 1.26 ^ab^
Irisin (pg/ml)	25.67 ± 1.1	16.33 ± 0.79 ^a^	20.75 ± 0.69 ^ab^	22.3 ± 0.96 ^b^	25.3 ± 1.1 ^bc^

Mean SE is the statistical distribution used to represent values. For each group, n is the total number of rats. A p-value < 0.05 was interpreted as demonstrating statistical significance

a significant when compared to control group at *P* < 0.05

b significant when compared to HCHFD group at *P* < 0.05

c significant when compared to AgNPs + HCHFD group at *P* < 0.05

d significant when compared to MO + HCHFD group at *P* < 0.05

The ability of MOs to improve insulin sensitivity, decrease leptin, and regulate metabolic abnormalities in obese rats was demonstrated by the overexpression of adiponectin and the downregulation of leptin in HCHFD-fed rats administered MO. Because adiponectin inhibits endothelial adhesion and suppresses the expression of “LDL scavenger receptors” on macrophages, it has anti-inflammatory properties that reduce the absorption of LDL and the development of plaque. Furthermore, when obesity and plasma insulin levels rise, visceral white adipose tissue rat adipocytes overexpress vaspin, a serine protease inhibitor produced by visceral adipose tissue. Therefore, MO’s capacity to raise insulin sensitivity was demonstrated by its ability to lower the amount of vaspin in adipose tissue [9].

The results of Ghezelbash et al. were consistent with the findings of our study, which showed that increasing fat mass in HCHFD-fed rats led to higher serum levels of resistin and leptin and lower levels of irisin and adiponectin [61]. The current study found that increasing fat mass promoted the synthesis of adipokines such as leptin [62] and resistin [61]. It also led to the activation of inflammatory cytokines. The observed decrease in food intake and the reported hyperleptinemia are suggestive of leptin resistance, a condition that led to decreased energy expenditure [63]. Insulin resistance development is directly correlated with resistin.

The results of our investigation showed that the HCHF diet group had the lowest serum irisin level, suggesting that a high-carbohydrate diet reduced irisin levels. These results are consistent with those of Kang et al. [64], who have demonstrated that irisin may be especially important for enhancing fat metabolism through exercise. This suggests that irisin may improve the energy consumption of adipose tissue [65].

According to Table 7, UCP1 and PPARGC1A significantly decreased in the HCHFD, AgNPs + HCHFD, and MO + HCHFD groups, whereas the MO-AgNPs + HCHFD group significantly increased in comparison to the control group. Additionally, in comparison to the HCHFD group, all treatment groups exhibited a substantial augmentation in the expressiveness of the UCP1 and PPARGC1A genes. However, there was no discernible difference between the groups that got MO + HCHFD and AgNPs + HCHFD. Conversely, the group that got the combination of *Moringa* and silver nanoparticles demonstrated a promising outcome, with their UCP1 and PPARGC1A levels nearly approaching the control values at *P* < 0.05 (Table 7).

**Table 7:** The UCP1 and PPARGC1A gene expression mRNA fold change measured by RT-qPCR in various groups

	Control *n* = 10	HCHFD *n* = 10	AgNPs + HCHFD *n* = 10	MO + HCHFD *n* = 10	MO-AgNPs + HCHFD *n* = 10
UCP1	1 ± 0.00	0.32 ± 0.04 ^acd^	0.81 ± 0.04 ^ab^	0.82 ± 0.04 ^ab^	1.29 ± 0.08 ^abcd^
PPARGC1A	1 ± 0.00	0.31 ± 0.02 ^acd^	0.77 ± 0.03 ^ab^	0.86 ± 0.03 ^ab^	1.52 ± 0.07 ^abcd^

Mean SE is the statistical distribution used to represent values. For each group, n is the total number of rats. A p-value < 0.05 was interpreted as demonstrating statistical significance

a significant when compared to control group at *P* < 0.05

b significant when compared to HCHFD group at *P* < 0.05

c significant when compared to AgNPs + HCHFD group at *P* < 0.05

d significant when compared to MO + HCHFD group at *P* < 0.05

From our data the expression of UCP1 gene is decreased in obesity. UCP1 is primarily expressed in brown adipose tissue (BAT), where it plays a critical role in the regulation of body weight, energy metabolism, thermogenesis, and glucose homeostasis. In obesity, the sympathetic nervous system activity is often reduced, and there is an increase in inflammatory cytokines, which can lead to a decrease in UCP1 expression in BAT. This reduction in UCP1 expression can lead to a decreased ability to generate heat through thermogenesis, which can contribute to weight gain and the onset of obesity. In addition to its role in thermogenesis, UCP1 has also been implicated in the regulation of glucose and lipid metabolism. Also, UCP1 activation can increase insulin sensitivity and improve glucose homeostasis, as well as decrease triglyceride levels in the liver and adipose tissue [66].

However, it is significant to remember that there are a number of complex elements that might affect the regulation of UCP1 expression in obesity, including hormone signaling pathways, genetics, and environmental factors. UCP1 expression can be increased in response to certain interventions, such as cold exposure or exercise, in both lean and obese individuals. Overall, while the expression of UCP1 gene is generally decreased in obesity, the regulation of UCP1 expression is intricate and susceptible to several factors [67].

The expression of PPARGC1A gene is reduce in obesity. PPARGC1A is a transcriptional coactivator that regulates mitochondrial biogenesis and oxidative metabolism in various tissues, including brown adipose tissue (BAT). In obesity, there is often a decrease in the expression of PPARGC1A due to insulin resistance and inflammation. This decrease in PPARGC1A expression can lead to a reduction in mitochondrial biogenesis and oxidative metabolism, which may have a role in the development of metabolic problems linked to obesity [68].

However, it is important to note that the regulation of PPARGC1A expression is complex and can be influenced by various factors, such as hormonal signaling pathways, genetic factors, and environmental factors. Overall, while the PPARGC1A gene expression was generally reduced in obesity, the regulation of PPARGC1A expression is complicated and subject to different influences.

**Fig. 4:**
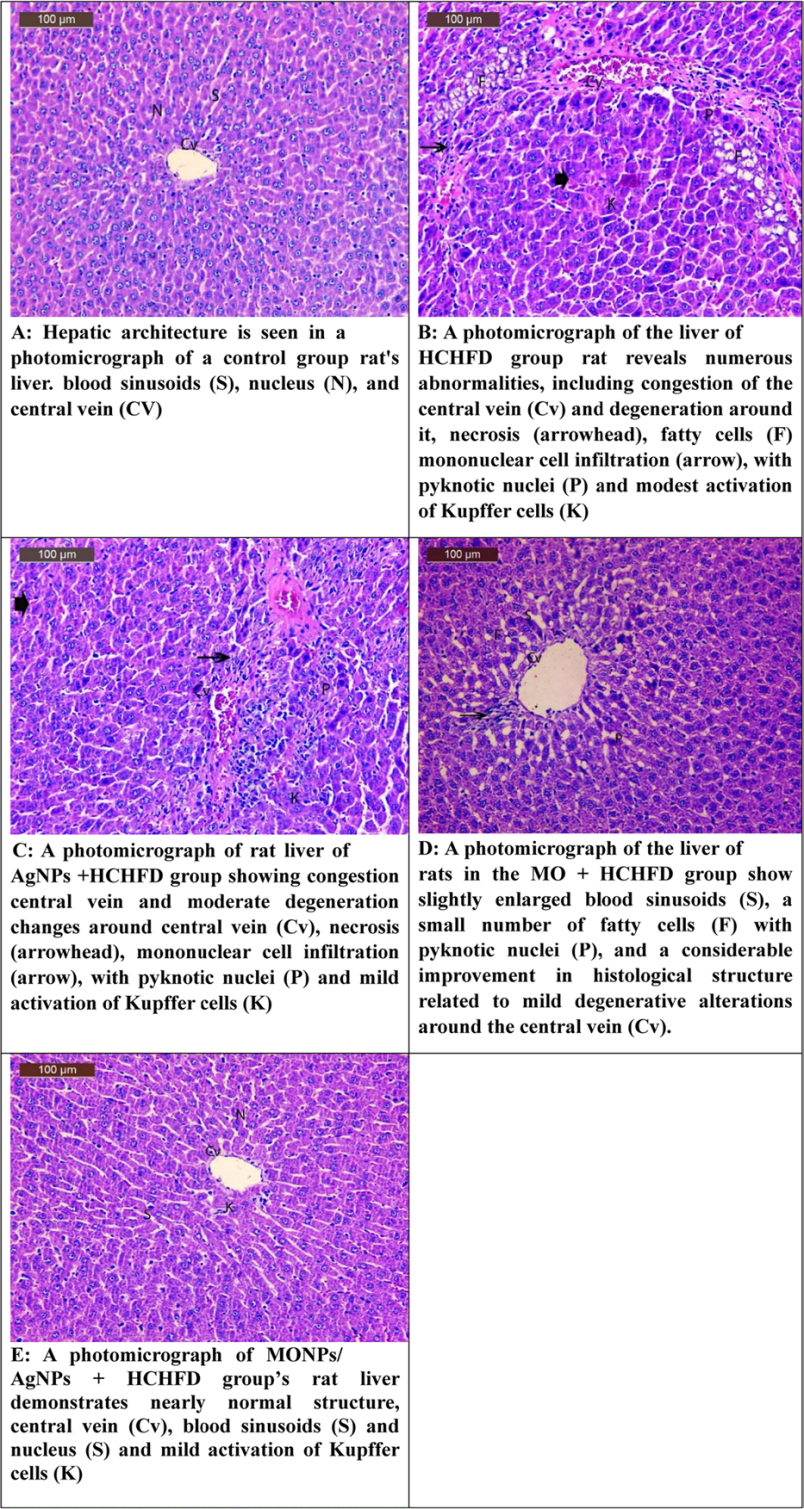
(**A**) Hepatic architecture is seen in a photomicrograph of a control group rat's liver. blood sinusoids (S), nucleus (N), and central vein (CV). (**B**) A photomicrograph of the liver of HCHFD group rat reveals numerous abnormalities, including congestion of the central vein (Cv) and degeneration around it, necrosis (arrowhead), fatty cells (F) mononuclear cell infiltration (arrow), with pyknotic nuclei (P) and modest activation of Kupffer cells (K). (**C**) A photomicrograph of rat liver of AgNPs +HCHFD group showing congestion central vein and moderate degeneration changes around central vein (Cv), necrosis (arrowhead), mononuclear cell infiltration (arrow), with pyknotic nuclei (P) and mild activation of Kupffer cells (K). (**D**) A photomicrograph of the liver of rats in the MO+ HCHFD group show slightly enlarged blood sinusoids (S), a small number of fatty cells (F) with pyknotic nuclei (P), and a considerable improvement in histological structure related to mild degenerative alterations around the central vein (Cv). (**E**) A photomicrograph of MONPs/ AgNPs + HCHFD group’s rat liver demonstrates nearly normal structure, central vein (Cv), blood sinusoids (S) and nucleus (S) and mild activation of Kupffer cells (K)

**Fig. 5:**
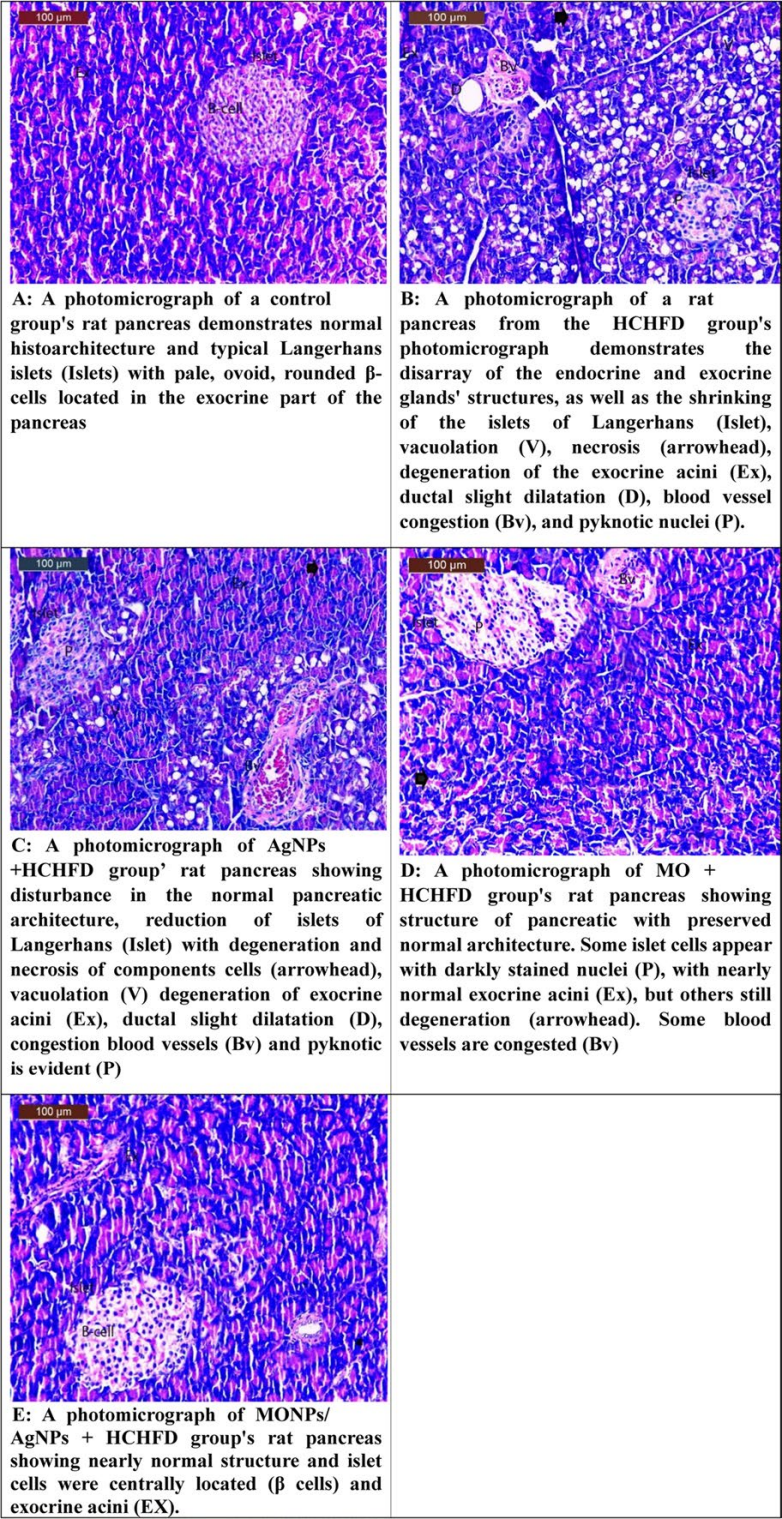
(**A**) A photomicrograph of a control group's rat pancreas demonstrates normal histoarchitecture and typical Langerhans islets (Islets) with pale, ovoid, rounded β-cells located in the exocrine part of the pancreas. (**B**) A photomicrograph of a rat pancreas from the HCHFD group's photomicrograph demonstrates the disarray of the endocrine and exocrine glands' structures, as well as the shrinking of the islets of Langerhans (Islet), vacuolation (V), necrosis (arrowhead), degeneration of the exocrine acini (Ex), ductal slight dilatation (D), blood vessel congestion (Bv), and pyknotic nuclei (P). (**C**) A photomicrograph of AgNPs +HCHFD group’ rat pancreas showing disturbance in the normal pancreatic architecture, reduction of islets of Langerhans (Islet) with degeneration and necrosis of components cells (arrowhead), vacuolation (V) degeneration of exocrine acini (Ex), ductal slight dilatation (D), congestion blood vessels (Bv) and pyknotic is evident (P) (**D**) A photomicrograph of MO + HCHFD group's rat pancreas showing structure of pancreatic with preserved normal architecture. Some islet cells appear with darkly stained nuclei (P), with nearly normal exocrine acini (Ex), but others still degeneration (arrowhead). Some blood vessels are congested (Bv). (**E**) A photomicrograph of MONPs/ AgNPs + HCHFD group's rat pancreas showing nearly normal structure and islet cells were centrally located (β cells) and exocrine acini (EX)

Histopathological analysis of the pancreas and liver tissues showed that the treatment groups’ cellular integrity and tissue architecture were significantly better than those of the high-carbohydrate high-fat diet (HCHFD) group. In particular, the MO-AgNPs therapy group showed significant restoration of normal cellular morphology, maintenance of lobular organization, and decreases in pancreatic and hepatic inflammation. In contrast to pancreatic acini, which displayed enhanced structural cohesiveness and less inflammatory cell infiltration, hepatocytes displayed less swelling and necrosis. These improvements in histology closely matched the biochemical results, indicating that MO-AgNPs may protect against pancreatic and liver damage brought on by diet. These findings are clearly supported by the photomicrographs that were supplied for each study group. These images demonstrate the therapeutic potential of MO-AgNPs in reducing the inflammatory and metabolic insults linked to obesity brought on by HCHFD (Fig. 4A-4E for liver and Fig. 5A-5E for pancreas).

## Conclusion

This study highlights the significant therapeutic potential of MO-AgNPs in addressing obesity and its related metabolic complications. A low oral dose of AgNPs (10 mg/kg/day) was used, which is generally considered safe in animal studies. Histopathological analysis showed reduced toxicity in the MO-AgNP treated group compared to AgNPs alone, suggesting that *Moringa* may mitigate the potential adverse effects of silver. The incorporation of silver nanoparticles enhanced the bioactivity of *Moringa Oleifera*, resulting in improved regulation of biochemical markers, lipid profiles, and adipokine levels, alongside upregulation of key thermogenic and metabolic genes. Moreover, histopathological evaluations confirmed the protective effects of MO-AgNPs on liver and pancreatic tissues, further supporting their role in mitigating obesity-induced organ damage. Importantly, *Moringa Oleifera* extracts, whether in their native form or encapsulated within AgNPs, demonstrated robust hypolipidemic properties, making them a versatile and effective option for combating obesity and improving overall metabolic health.

## Supplementary Information

Below is the link to the electronic supplementary material.

Supplementary Material 1 (DOCX 149 KB)

## Data Availability

The article/supplementary material contains the original contributions made during the study; for more information, contact the corresponding author or authors.

## Abbreviations

AgNPs: Silver Nanoparticles
UCP1: Uncoupling protein-1
TEM: Transmission electron microscopy
PPARGC1A: Peroxisome proliferator activated receptor gamma co-activator 1alpha
ELISA: Enzyme linked immunosorbent assay
HPLC: High-performance liquid chromatography
DLS: Dynamic light scattering
MO: Moringa Oleifera
RT-PCR: Real-time polymerase chain reaction
SE: Standard error
SPSS: Statistical package for the social science
NaOH: Sodium hydroxide
WAT: White adipose tissue
ATP: Adenosine triphosphate
FXR: Farnesoid X receptor
VLDL: Very low -density lipoprotein
HNF4α: Hepatocyte nuclear factor 4α

## References

[1] Tammam, M. A., et al. (2024). Unveiling the potential of marine-derived diterpenes from the order alcyonacea as promising anti-obesity agents. *Current Research in Biotechnology*, *7*, 100175.

[2] Aly, O., et al. (2020). Gene polymorphisms of Patatin-like phospholipase domain containing 3 (PNPLA3), adiponectin, leptin in diabetic obese patients. *PLoS One,**15*(6), e0234465. 32544194 10.1371/journal.pone.0234465 PMC7297308

[3] Dri, M., Klinger, F. G., & De Felici, M. (2021). The ovarian reserve as target of insulin/IGF and ROS in metabolic disorder-dependent ovarian dysfunctions. *Reproduction and Fertility*, *2*(3), R103–R112. 35118400 10.1530/RAF-21-0038 PMC8801032

[4] Takashima, S., et al. (2019). Concentrations of leptin, adiponectin, and resistin in the serum of obese cats during weight loss. *Journal of Veterinary Medical Science*, *81*(9), 1294–1300. 31366817 10.1292/jvms.19-0091 PMC6785622

[5] Tong, Y., et al. (2022). Obesity and insulin resistance: Pathophysiology and treatment. *Drug Discovery Today*, *27*(3), 822–830. 34767960 10.1016/j.drudis.2021.11.001

[6] Mesri Alamdari, N., et al. (2020). Effects of Royal jelly and Tocotrienol rich fraction in obesity treatment of calorie-restricted obese rats: A focus on white fat Browning properties and thermogenic capacity. *Nutrition & Metabolism*, *17*, 1–13. 32508963 10.1186/s12986-020-00458-8 PMC7266117

[7] Bhatta, P., et al. (2020). Meta-analysis demonstrates Gly482Ser variant of PPARGC1A is associated with components of metabolic syndrome within Asian populations. *Genomics*, *112*(2), 1795–1803. 31678594 10.1016/j.ygeno.2019.10.011

[8] Fan, X., et al. (2020). High-fat diet alters the expression of reference genes in male mice. *Frontiers in Nutrition*, *7*, 589771. 33330591 10.3389/fnut.2020.589771 PMC7732482

[9] Ndlovu, S. S., Ghazi, T., & Chuturgoon, A. A. (2022). The potential of *Moringa oleifera* to ameliorate HAART-induced pathophysiological complications. *Cells,**11*(19), 2981. 36230942 10.3390/cells11192981 PMC9563018

[10] Mehanna, E. T., et al. (2022). Anti-oxidant, anti-apoptotic, and mitochondrial regulatory effects of selenium nanoparticles against Vancomycin induced nephrotoxicity in experimental rats. *Life Sciences*, *288*, 120098. 34715137 10.1016/j.lfs.2021.120098

[11] Shoman, N. A., et al. (2025). LC-MS/MS profiling and Immunomodulatory potential of green-synthesized zinc oxide nanoparticles using cordia Sebestena leaves against chromium-induced acute lung injury in rats. *Journal of Drug Delivery Science and Technology*, *106*, 106750.

[12] Younis, N., et al. (2022). Phytochemical and antioxidant screening of *Moringa oleifera* for its utilization in the management of hepatic injury. *Frontiers in Nutrition,**9*, 1078896. 36590207 10.3389/fnut.2022.1078896 PMC9797499

[13] Unhapipatpong, C., et al. (2023). The effect of Curcumin supplementation on weight loss and anthropometric indices: An umbrella review and updated meta-analyses of randomized controlled trials. *American Journal of Clinical Nutrition,**117*(5), 1005–1016. 36898635 10.1016/j.ajcnut.2023.03.006

[14] Mongioì, L. M., et al. (2021). The role of Resveratrol administration in human obesity. *International Journal of Molecular Sciences*, *22*(9), 4362. 33921991 10.3390/ijms22094362 PMC8122246

[15] Suzuki, T., et al. (2016). Beneficial effects of tea and the green tea Catechin epigallocatechin-3-gallate on obesity. *Molecules*, *21*(10), 1305. 27689985 10.3390/molecules21101305 PMC6274011

[16] Cortes-Alvarez, S. I., et al. (2024). Efficacy of hot tea infusion vs. Ethanolic extract of Moringa Oleifera for the simultaneous treatment of nonalcoholic fatty Liver, Hyperlipidemia, and hyperglycemia in a murine model fed with a High-Fat diet. *Journal of Nutrition and Metabolism*, *2024*(1), 2209581. 38375319 10.1155/2024/2209581 PMC10876314

[17] Redha, A. A., et al. (2021). Novel insights on anti-obesity potential of the miracle tree, Moringa oleifera: A systematic review. *Journal of Functional Foods*, *84*, 104600.

[18] Sofini, P. S., et al. (2024). Evaluation of scarless wound healing through nanohydrogel infused with selected plant extracts. *Journal of Drug Delivery Science and Technology*, *100*, 106118.

[19] Paul, S., et al. (2024). Silver nanoparticles in diabetes mellitus: Therapeutic potential and mechanistic insights. *Bulletin of the National Research Centre*, *48*(1), 33.

[20] Madbouly, N., et al. (2025). Green silver nanoparticles ameliorate diet-induced obesity through antioxidant and anti-inflammatory properties. *Beni-Suef University Journal of Basic and Applied Sciences*, *14*(1), 1–23.

[21] Dinç, B. (2025). Comprehensive toxicity assessment of silver nanoparticles on Bacteria, human vein endothelial cells, and caenorhabditis elegans. *Results in Chemistry*, *14*, 102092.

[22] Ahmed, S., et al. (2016). Green synthesis of silver nanoparticles using Azadirachta indica aqueous leaf extract. *Journal of Radiation Research and Applied Sciences*, *9*(1), 1–7.

[23] Sharma, N. K., et al. (2022). Green route synthesis and characterization techniques of silver nanoparticles and their biological adeptness. *ACS Omega*, *7*(31), 27004–27020. 35967040 10.1021/acsomega.2c01400 PMC9366950

[24] Hossain, N., Islam, M. A., & Chowdhury, M. A. (2022). *S*ynthesis and characterization of plant extracted silver nanoparticles and advances in dental implant applications. *Heliyon*, *8*(12). 10.1016/j.heliyon.2022.e12313 PMC9794905 36590472

[25] Mohammed, G. M., & Hawar, S. N. (2022). Green biosynthesis of silver nanoparticles from Moringa Oleifera leaves and its antimicrobial and cytotoxicity activities. *International Journal of Biomaterials*, *2022*(1), 4136641. 36193175 10.1155/2022/4136641 PMC9526645

[26] Hussein, J., et al. (2019). Synthesis of docosahexaenoic acid–loaded silver nanoparticles for improving endothelial dysfunctions in experimental diabetes. *Human & Experimental Toxicology*, *38*(8), 962–973. 31018711 10.1177/0960327119843586

[27] Yakout, S. M., & Mostafa, A. A. (2015). A novel green synthesis of silver nanoparticles using soluble starch and its antibacterial activity. *International Journal of Clinical and Experimental Medicine*, *8*(3), 3538. 26064246 PMC4443080

[28] Liaqat, N., et al. (2022). Green synthesized silver nanoparticles: Optimization, characterization, antimicrobial activity, and cytotoxicity study by hemolysis assay. *Frontiers in Chemistry*, *10*, 952006. 36105303 10.3389/fchem.2022.952006 PMC9465387

[29] Reeves, P. G. (1997). Components of the AIN-93 diets as improvements in the AIN-76A diet. *The Journal of Nutrition*, *127*(5), 838S–841S. 9164249 10.1093/jn/127.5.838S

[30] Bais, S., Singh, G. S., & Sharma, R. (2014). Antiobesity and hypolipidemic activity of Moringa Oleifera leaves against high fat diet-induced obesity in rats. *Advances in Biology*, *2014*(1), 162914.

[31] El-Bakry, K. Protective Effects of Silver Nanoparticles of Moringa Oleifera Leaves against Acrylamide-Induced Blood Toxicity in Rats.

[32] Passing, H., & Bablok, W. (1983). A new biometrical procedure for testing the equality of measurements from two different analytical methods*. Application of linear regression procedures for method comparison studies in clinical chemistry*. *Part I*. 10.1515/cclm.1983.21.11.709 6655447

[33] Berson, S. A., & Yalow, R. S. (1961). Plasma insulin in health and disease. *The American Journal of Medicine*, *31*(6), 874–881. 13868361 10.1016/0002-9343(61)90029-8

[34] Matthews, D. R., et al. (1985). Homeostasis model assessment: Insulin resistance and β-cell function from fasting plasma glucose and Insulin concentrations in man. *Diabetologia*, *28*, 412–419. 3899825 10.1007/BF00280883

[35] Richmond, W. (1973). Preparation and properties of a cholesterol oxidase from nocardia sp. and its application to the enzymatic assay of total cholesterol in serum. *Clinical Chemistry*, *19*(12), 1350–1356. 4757363

[36] Cole, T., et al. (1984). Effects of high cholesterol diets on rat plasma lipoproteins and lipoprotein-cell interactions. *Journal of Lipid Research*, *25*(6), 593–603. 6431045

[37] Lopes-Virella, M. F., et al. (1977). Cholesterol determination in high-density lipoproteins separated by three different methods. *Clinical Chemistry*, *23*(5), 882–884. 192488

[38] Friedewald, W. T., Levy, R. I., & Fredrickson, D. S. (1972). Estimation of the concentration of low-density lipoprotein cholesterol in plasma, without use of the preparative ultracentrifuge. *Clinical Chemistry*, *18*(6), 499–502. 4337382

[39] Livak, K. J., & Schmittgen, T. D. (2001). Analysis of relative gene expression data using real-time quantitative PCR and the 2 – ∆∆CT method. *Methods*, *25*(4), 402–408. 11846609 10.1006/meth.2001.1262

[40] Aly, O., et al. (2020). Hepatoprotective effect of *Moringa oleifera* extract on TNF-α and TGF-β expression in acetaminophen-induced liver fibrosis in rats. *Egyptian Journal of Medical Human Genetics,**21*, 1–9.

[41] Anigol, L. B., Charantimath, J. S., & Gurubasavaraj, P. M. (2017). Effect of concentration and pH on the size of silver nanoparticles synthesized by green chemistry. *Org Med Chem Int J*, *3*(5), 1–5.

[42] Joshi, S. J. (2018). Green synthesis of silver nanoparticles using pomegranate Peel extracts and its application in photocatalytic degradation of methylene blue. *Jundishapur Journal of Natural Pharmaceutical Products*, *13*(3).

[43] Singh, M., Sinha, I., & Mandal, R. (2009). Role of pH in the green synthesis of silver nanoparticles. *Materials Letters*, *63*(3–4), 425–427.

[44] Sathishkumar, M., et al. (2009). *Cinnamon zeylanicum* bark extract and powder mediated green synthesis of nano-crystalline silver particles and its bactericidal activity. *Colloids and Surfaces B: Biointerfaces,**73*(2), 332–338. 19576733 10.1016/j.colsurfb.2009.06.005

[45] Asimuddin, M., et al. (2020). *Azadirachta indica* based biosynthesis of silver nanoparticles and evaluation of their antibacterial and cytotoxic effects. *Journal of King Saud University-Science,**32*(1), 648–656.

[46] Alafandi, L., et al. (2021). Green synthesis of silver nanoparticles using coffee extract for catalysis. *Malaysian NANO-An International Journal*, *1*(2), 13–25.

[47] Kredy, H. M. (2018). The effect of pH, temperature on the green synthesis and biochemical activities of silver nanoparticles from lawsonia inermis extract. *Journal of Pharmaceutical Sciences and Research*, *10*(8), 2022–2026.

[48] Stavinskaya, O., et al. (2019). Effect of temperature on green synthesis of silver nanoparticles using *vitex agnus-castus* extract. *Chemistry Journal of Moldova,**14*(2), 117–121.

[49] Chandra, H., et al. (2025). Eco-friendly silver nanoparticles synthesis method using medicinal plant fungal endophytes—Biological activities and molecular docking analyses. *Biology,**14*(8), 950. 40906197 10.3390/biology14080950 PMC12383769

[50] El-Naggar, N. E. A., et al. (2024). Myco-biosynthesis of silver nanoparticles, optimization, characterization, and in silico anticancer activities by molecular docking approach against hepatic and breast cancer. *Biomolecules,**14*(9), 1170. 39334936 10.3390/biom14091170 PMC11429812

[51] He, Y., et al. (2017). Green synthesis of silver nanoparticles using seed extract of *Alpinia katsumadai*, and their antioxidant, cytotoxicity, and antibacterial activities. *RSC Advances,**7*(63), 39842–39851.

[52] Kilany, O. E., et al. (2020). Anti-obesity potential of *Moringa olifera* seed extract and lycopene on high fat diet induced obesity in male sprauge Dawely rats. *Saudi Journal of Biological Sciences,**27*(10), 2733–2746. 32994733 10.1016/j.sjbs.2020.06.026 PMC7499387

[53] Imran, M., et al. (2022). Therapeutic application of carvacrol: A comprehensive review. *Food Science & Nutrition*, *10*(11), 3544–3561. 36348778 10.1002/fsn3.2994 PMC9632228

[54] Al-Malki, A. L., & Rabey, H. A. E. (2015). The antidiabetic effect of low doses of Moringa Oleifera Lam. Seeds on streptozotocin induced diabetes and diabetic nephropathy in male rats. *BioMed Research International*, *2015*(1), 381040. 25629046 10.1155/2015/381040 PMC4299558

[55] Alkhalidy, H., et al. (2018). Kaempferol ameliorates hyperglycemia through suppressing hepatic gluconeogenesis and enhancing hepatic insulin sensitivity in diet-induced obese mice. *The Journal of Nutritional Biochemistry*, *58*, 90–101. 29886193 10.1016/j.jnutbio.2018.04.014 PMC6095729

[56] Toma, A. (2022). Antidiabetic activity of hot tea infusion of leaves of Moringa stenopetala in streptozotocin-induced diabetic rats. *Journal of Experimental Pharmacology*, 309–316. 10.2147/JEP.S371354 PMC9617514 36317069

[57] Vogiatzi, G., Tousoulis, D., & Stefanadis, C. (2009). The role of oxidative stress in atherosclerosis. *Hellenic Journal of Cardiology,**50*(5), 402–409. 19767282

[58] Villarruel-López, A., et al. (2018). Effect of *Moringa oleifera* consumption on diabetic rats. *BMC Complementary and Alternative Medicine,**18*, 1–10. 29636032 10.1186/s12906-018-2180-2 PMC5894151

[59] Yassa, H. D., & Tohamy, A. F. (2014). Extract of *Moringa oleifera* leaves ameliorates streptozotocin-induced diabetes mellitus in adult rats. *Acta Histochemica,**116*(5), 844–854. 24657072 10.1016/j.acthis.2014.02.002

[60] Salem, H. F., et al. (2022). Rosuvastatin calcium-based novel nanocubic vesicles capped with silver nanoparticles-loaded hydrogel for wound healing management: Optimization employing Box–Behnken design: In vitro and in vivo assessment. *Journal of Liposome Research*, *32*(1), 45–61. 33353435 10.1080/08982104.2020.1867166

[61] Ghezelbash, B., et al. (2022). Protective roles of Shilajit in modulating resistin, adiponectin, and cytokines in rats with non-alcoholic fatty liver disease. *Chinese Journal of Integrative Medicine*, *28*(6), 531–537. 35258780 10.1007/s11655-022-3307-3

[62] Saucillo, D. C., et al. (2014). Leptin metabolically licenses T cells for activation to link nutrition and immunity. *The Journal of Immunology*, *192*(1), 136–144. 24273001 10.4049/jimmunol.1301158 PMC3872216

[63] Saxton, R. A., et al. (2023). Structural insights into the mechanism of leptin receptor activation. *Nature Communications*, *14*(1), 1797. 37002197 10.1038/s41467-023-37169-6 PMC10066393

[64] Kang, Y. S., et al. (2019). Effects of swimming exercise on serum Irisin and bone FNDC5 in rat models of high-fat diet-induced osteoporosis. *Journal of Sports Science & Medicine*, *18*(4), 596. 31827343 PMC6873128

[65] Waseem, R., et al. (2022). FNDC5/irisin: Physiology and pathophysiology. *Molecules*, *27*(3), 1118. 35164383 10.3390/molecules27031118 PMC8838669

[66] Maliszewska, K., & Kretowski, A. (2021). Brown adipose tissue and its role in insulin and glucose homeostasis. *International Journal of Molecular Sciences*, *22*(4), 1530. 33546400 10.3390/ijms22041530 PMC7913527

[67] Schirinzi, V., et al. (2023). Browning of adipocytes: A potential therapeutic approach to obesity. *Nutrients*, *15*(9), 2229. 37432449 10.3390/nu15092229 PMC10181235

[68] Huang, M., et al. (2023). Engineered allele substitution at PPARGC1A rs8192678 alters human white adipocyte differentiation, lipogenesis, and PGC-1α content and turnover. *Diabetologia*, *66*(7), 1289–1305. 37171500 10.1007/s00125-023-05915-6 PMC10244287

