# Supplementary figures and images for: Modulatory Role of Moringa Oleifera-Loaded Silver Nanoparticles on UCP1 and PPARGC1A Genes Expression in an Obesity Rat Model
Source: Appl Biochem Biotechnol. 2026 Jan 23;198(4):2683–707. doi: 10.1007/s12010-025-05511-x (PMC13032946; doi:10.1007/s12010-025-05511-x)

| 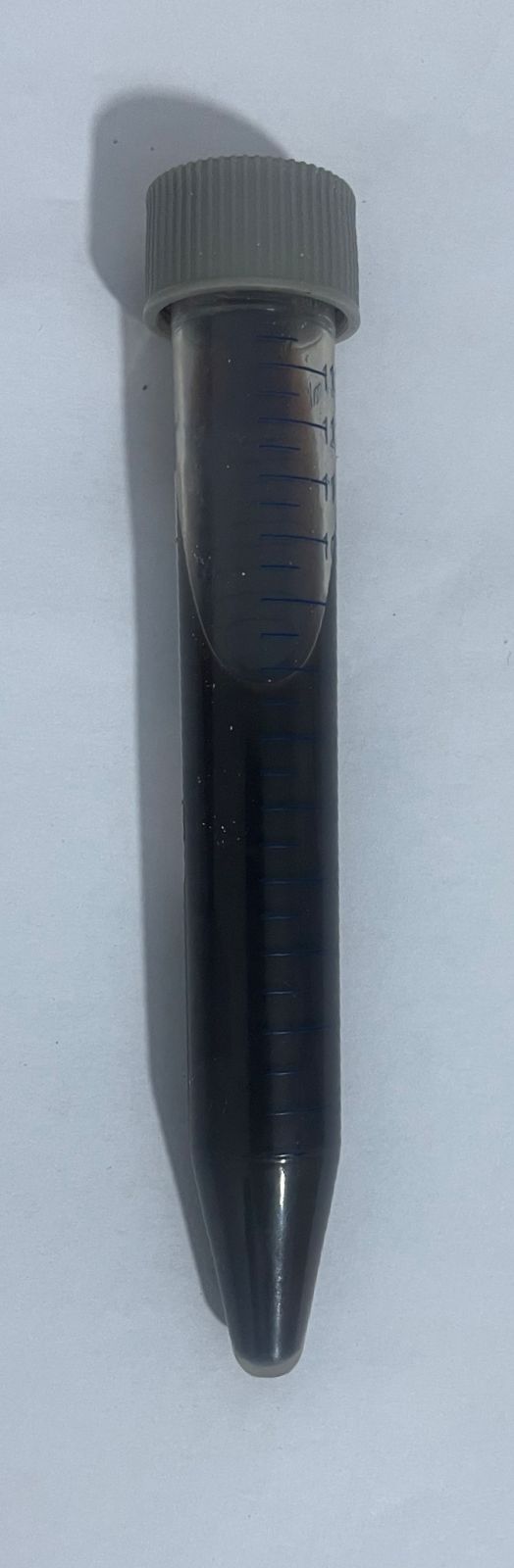 | 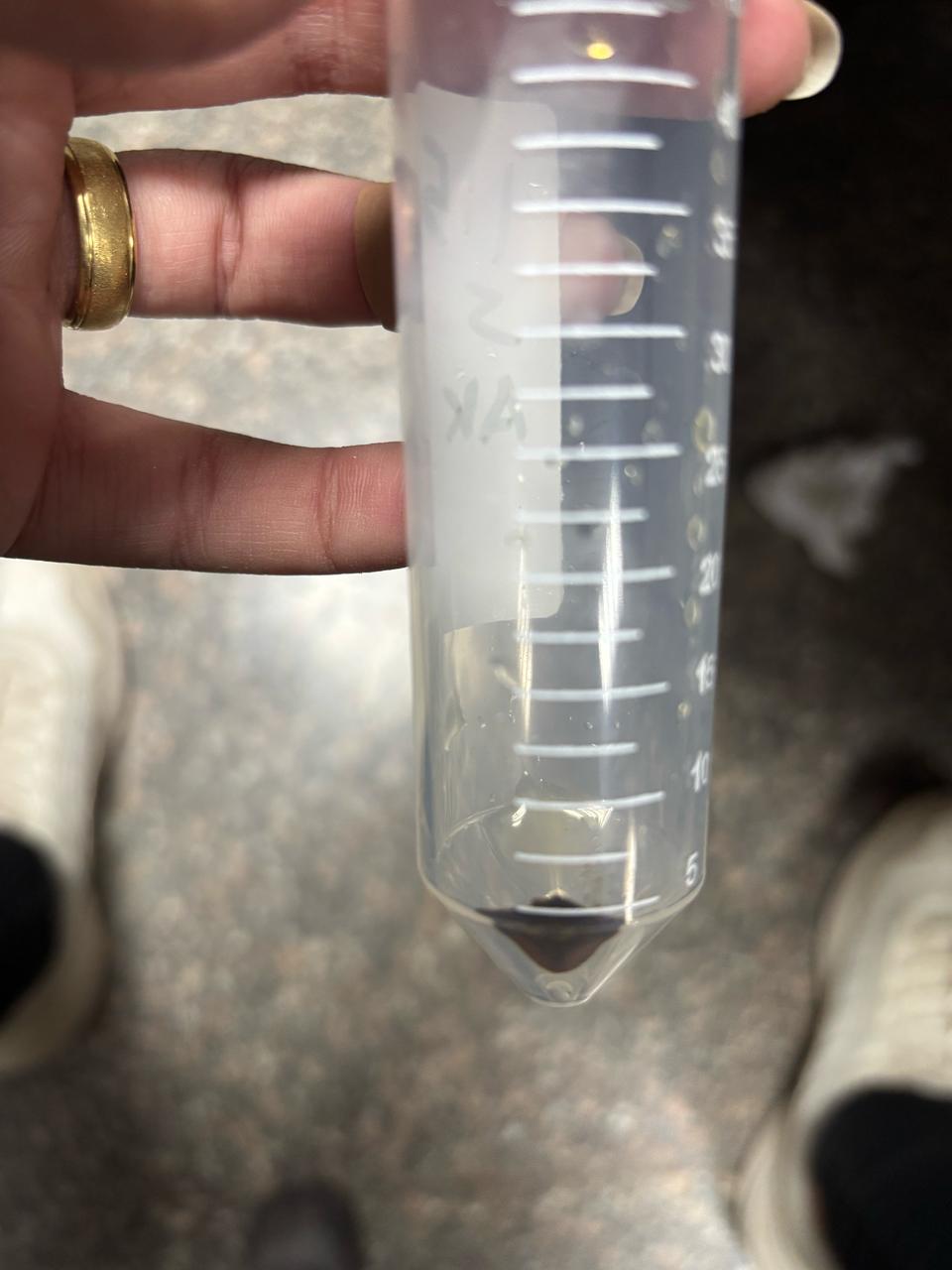 |
| --- | --- |
| **Silver Nanoparticles** | **Silver Nanoparticles Powders (After Centrifugation)** |

Supplement: Supplementary file 1 — Supplementary Material 1 (DOCX 149 KB) [file 12010_2025_5511_MOESM1_ESM.docx]
